# IAB 17th World Congress

**DOI:** 10.1111/bioe.70066

**Published:** 2025-12-07

**Authors:** Cristina Richie

**Affiliations:** ^1^ The University of Edinburgh Edinburgh Scotland UK

The 17th World Congress of Bioethics was held at the Qatar National Convention Center, Doha from June 03‐06, 2024. It was a hybrid event, with 855 IAB registrants and 62 FAB registrants from 74 countries. The Congress was organized by the Center for Islamic Legislation and Ethics (CILE) and World Innovation Summit for Health (WISH) [1]. The theme was “Religion, Culture and Global Bioethics” but, of course, there were numerous topics around 10 key themes:

1‐ Armed Conflict, War and Healthcare
2‐ Artificial Intelligence in Healthcare
3‐ Disability Bioethics
4‐ Environmental Bioethics
5‐ Genetic/Genomic Ethics (GenEthics)
6‐ Interplay of Culture and Bioethics
7‐ Key Bioethical Concepts: Cultural, Religious, and Secular Perspectives
8‐ Pandemics and COVID‐19: Public Health Ethics
9‐ Religious Traditions and Bioethical Reasoning
10‐ Teaching Bioethics Across Different Cultures and Moral Traditions

Prior to the event, the location of the Congress spurred formal and informal debate around what may be called “cooperation with” or “proximity to” evil. The Catholic Catechism outlines one of the best examples of how to navigate the moral conflict of being associated with ethically problematic people or practices, when one is not, themselves, doing any wrong. Pope John Paul II writes, “we have a responsibility for the sins committed by others when we cooperate in them:

− by participating directly and voluntarily in them;
− by ordering, advising, praising, or approving them;
− by not disclosing or not hindering them when we have an obligation to do so;
− by protecting evil‐doers.” [2]

Of the many “sins,” Van der Graaf, Jongsma, van de Vathorst, de Vries and Bolt point out, “Qatar is known for violations of human rights—including the treatment of migrant workers and the rights of women—corruption, criminalization of LGBTQI+ persons, and climate impact.” [3] Of course, the practices and policies of a government are different than the many people of goodwill inhabiting that country, as global events—from US politics to the war in Palestine and Ukraine—attest to.

However, the rationale for selecting Qatar as a location for the Congress was distinct from the above concerns. Udo Schuklenk's editorial noted that the IAB chose Qatar based on the criteria of ability to provide scholarships—especially people from the Global South—the geographical advantage for those in the Middle East and Asia, and the historical choice to host a World Congress in a majority Muslim country [4].

Many IAB and FAB members felt the weight of this choice. And, unlike other Congresses, each person was confronted with the ethical ambiguities and make a decision—that conformed to their conscience—if they would participate or not.

In some cases, any connection with Qatar, from travel—which would be carbon intensive and fund a corrupt government—to tourist activities were viewed as direct participation in nefarious activities (the first clause of the catechism); others saw more distance between the Congress and the country. But, in all cases, there was resounding consensus that participation in this Congress was not approval of the government (the second clause of the catechism).

All three of the formal responses to the editorial in *Bioethics* 37 no. 4 and the follow up letters and responses in the same volume and 38, no 7 (one of which was mine, for transparency) resoundingly exposed, and refused to protect evil‐doers (the third and fourth clauses of the catechism). It is against this impassioned background that papers were submitted for the *Special Issue* and the selections made through the peer review process.

I am grateful to my coeditor Ruth Chadwick for her wisdom and efficiency; Ms. Clancy Pegg, the Managing Editor of *Bioethics,* for being organized and diligent in keeping track of papers and contacting reviewers; the reviewers who generously offered feedback and of course, all the authors who submitted their papers for consideration in this issue.

The first four papers are organized around the theme of ethical frameworks and principles. Appropriate for the context of the Congress, the first two articles address political aspects of bioethics and the extent to which human life and health intersect with disruptive or destructive politics. Annoni examines political legitimacy while Lederman focuses on Gaza. The next two articles have notions of human‐centric medicine at their core but offer very different scopes, with Saleem working on autonomy and Kunda‐Ng'andu and Muleba on community. Thematically, this first set of papers underscores interconnection and the moral obligations we have to each other as “our sister's keeper.” Humankind lives intertwined, our actions as individuals, as health care providers, as ethicists, even as politicians have ripple effects. These effects may break—or heal—others. The choice is ours.

The next two papers continue in the vein of ethical frameworks, but from monotheistic traditions. The third largest number of submissions to the Congress were to the *Religious Traditions and Bioethical Reasoning* theme. And, in keeping with one of the more prominent features of the Congress, the paper from Muhsin, Hashi, Gounjaria, and Chin offer an illuminating essay on Islamic jurisprudence, while the paper from Roman excavates the relevance of Orthodox Christianity for bioethical issues. Though based in faith traditions, we should observe that one's faith (or lack thereof) may or may not be represented in written scholarship. As Jacques Maritain notes “to speak as a Catholic having certain temporal position and to speak in the name of Catholicism are two very different things.” [5] Thus, this set of articles gives us an invitation to explore and appreciate the unique dimensions of particular religious tradition without having to assent to their cosmological commitment.

The next part of the journal moves from ethical frameworks to specific topics in biomedical ethics. Three essays address the high‐interest topic of artificial intelligence (AI), observing the numerous challenges—and possibilities of the technology. Padela, Hayek, Tabassum, Jotter, and and Qadir offer a wide overview of AI and associated technologies, inspecting how they can be used ethically in health care. AI technologies, of course, are directly tied to machine learning and algorithms. These algorithms can simplify complex tasks, but are associated with a number of concerns, such as “black box” decision making, which Allen, Wilkinson, and Savulescu address. A similar, and related, concern is algorithmic bias; how this intersects with AI in health care is the topic of the third article in this set, by Anantharaman, Savulescu, and Schaefer. AI will certainly continue to be a theme in biomedical ethics and exploring the disciplines of both ethics of technology and biomedical ethics will provide ethicists no shortage of work in the future.

Beyond the AI revolution, the last two sets of papers address more perennial bioethical concerns: beginning‐of‐life and end‐of‐life. The first essay of the set, from Ong, places Singapore as the cultural setting for an exploration of the responsibility narrative in familial genetic risk discussions. Döbler, Pastukhov, and Carbon use social science research to examine hypothetical impacts of genetic engineering on ethnicity. Finally, Blevennec makes the ethical distinction between genetic counselling and genetic testing, arguing that the former should be directive and the later non‐directive. Contextualization is key in reproductive and genetic ethics.

Similarly, the final set of papers on end‐of‐life are bounded within specific cultures. Hassanein's advocacy for empowering Muslim patients and families in Canada in end‐of‐life decision making offers a valuable contribution to culturally responsive biomedical ethics. Chung reevaluates the idea of a “Good Death” in Taiwan, giving the readers a concise understanding of the deceased organ legislation there. And, in the last essay, Yazici and Elsebahy offer ethical reflection at the intersection of Islam and organ bioprinting.

The Presidential address from Dr. Caesar Atuire, rounds out and complements many of the themes outlined in this issue. It can be watched on the Congress website [6]. He effortlessly manages to harvest the wisdom of history and scan the future for a path toward pluriversality. In doing so he also mirrors the essence of the 17th World Congress of Bioethics, navigating, like a sailor, the numerous challenges to bioethics, human dignity, and solidarity. Though the skies be dark, the stars are brilliant.
